# Protein network analysis to prioritize key genes in amyotrophic lateral sclerosis

**DOI:** 10.1016/j.ibneur.2021.12.002

**Published:** 2021-12-07

**Authors:** Rupesh Kumar, Shazia Haider

**Affiliations:** Department of Biotechnology, Jaypee Institute of Information Technology, Noida, Sec-62, Uttar Pradesh, India

**Keywords:** ALS, Amyotrophic Lateral Sclerosis, ALSoD, Amyotrophic Lateral Sclerosis online database, ALS-PPIN, Amyotrophic Lateral Sclerosis Protein-Protein Interaction Network, BC, Betweenness centrality, Bn-H, Bottleneck-hub, CDC5L, Cell division cycle5-likeprotein, FUS, Fused in sarcoma, MND, Motor neuron disease, MCODE, Molecular Complex Detection, SOD1, Superoxide dismutase, SNW1, SNW domain-containing protein 1, SMA, Spinal muscular atrophy, SMN, Survival of motor neuron, TP53, Tumor protein p53, VCP, Valosin containing protein, ALS, ALS-PPIN, *CDC5L*, *SNW1*, *TP53*, *SOD1*, *VCP*, Bottleneck-hubs

## Abstract

Amyotrophic Lateral Sclerosis (ALS) is a fatal disease, progressive nature characterizes by loss of both upper and lower motor neuron functions. One of the major challenge is to understand the mechanism of ALS multifactorial nature. We aimed to explore some key genes related to ALS through bioinformatics methods for its therapeutic intervention. Here, we applied a systems biology approach involving experimentally validated 148 ALS-associated proteins and construct ALS protein-protein interaction network (ALS-PPIN). The network was further statistically analysed and identified bottleneck-hubs. The network is also subjected to identify modules which could have similar functions. The interaction between the modules and bottleneck-hubs provides the functional regulatory role of the ALS mechanism. The ALS-PPIN demonstrated a hierarchical scale-free nature. We identified 17 bottleneck-hubs, in which *CDC5L*, *SNW1, TP53, SOD1,* and *VCP* were the high degree nodes (hubs) in ALS-PPIN. *CDC5L* was found to control highly cluster modules and play a vital role in the stability of the overall network followed by *SNW1*, *TP53, SOD1,* and *VCP*. *HSPA5 and HSPA8 acting* as a common connector for *CDC5L* and *TP53* bottleneck-hubs. The functional and disease association analysis showed ALS has a strong correlation with mRNA processing, protein deubiquitination, and neoplasms, nervous system, immune system disease classes. In the future, biochemical investigation of the observed bottleneck-hubs and their interacting partners could provide a further understanding of their role in the pathophysiology of ALS.

## Highlights

• Amyotrophic Lateral Sclerosis protein-protein interaction network (ALS-PPIN) followed a hierarchical scale-free nature.
• We identified 17 bottleneck-hubs in the ALS-PPIN.
• Among bottleneck-hubs we found CDC5L, SNW1, TP53, SOD1, and VCP were the high degree nodes (hubs) in the ALS-PPIN.
• CDC5L is the effective communicator with all five modules in the ALS-PPIN and followed by SNW1 and TP53.
• Modules are highly associated with various disease classes like neoplasms, nervous systems and others.

## 1 Introduction

Amyotrophic lateral sclerosis (ALS) is one of the fatal neurodegenerative disease mainly occurs due to the loss of motor neuron and neuronal death (Martin et al., 2017). It leads to death by progressive paralysis and respiratory failure within 2–4 years(Chio et al., 2009). About 90–95% of ALS cases are sporadic (sALS) and 5–10% are inherited through family (fALS)(Van Rheenen et al., 2016). Many genetic factors have been identified, involving aggravations for RNA metabolism(Butti and Patten, 2018), weakened protein homeostasis(Cykowski et al., 2019), damaged DNA repair (Brenner et al., 2016), disruption of nucleocytoplasmic transport (Kim and Taylor, 2017), excitotoxicity(Foerster et al., 2013), oxidative stress(Mitsumoto et al., 2008), and axonal transport disturbance(De Vos et al., 2007). Mutations observed in several genes related to ALS such as *SOD1*(Rosen et al., 1993), *FUS* (Kwiatkowski et al., 2009), *C9ORF72* (DeJesus-Hernandez et al., 2011, Renton et al., 2011), *ATXN2* (Elden et al., 2010), *OPTN* (Maruyama et al., 2010), *VCP* (Johnson et al., 2010), *PFN1* (Wu et al., 2012), *MATR3* (Johnson et al., 2014), *SETX* (Hirano et al., 2011), *UBQLN2* (Deng et al., 2011). Initial discovery of a risk associated *C9ORF72* locus in ALS (GWAS), a pathology associated risk hexanucleotide-repeat expansion (G4C2) updated the field of ALS genetics and biology (Laaksovirta et al., 2010). Since 2015, others gene such as *TBK1*(Freischmidt et al., 2015)*, C21ORF2*(Van Rheenen et al., 2016)*, NEK1* (Brenner et al., 2016*), CCNF*(Williams et al., 2016)*, MOBP,SCFD1*(Van Rheenen et al., 2016)*, KIF5A*(Nicolas et al., 2018)*, LGALSL* (Gelfman et al., 2019)*, GLT8D1* (Cooper-Knock et al., 2019)*, DNAJC7*(Farhan et al., 2019)*, TUBA4A*(Smith et al., 2014)*, ANXA11*(Topp et al., 2017)*, UBQLN4* (Edens et al., 2017)*, CHCHD10* (Woo et al., 2017) identified through GWAS, whole-genome and exome sequencing methods correlated with ALS. The genes like *SOD1, C9ORF72, FUS and TDP-43* are highly mutated as compared to other ALS associated genes and mostly associated to ROS-associated oxidative stress, excitotoxicity, protein aggregation, altered RNA processing, axonal and vesicular trafficking dysregulation, and mitochondrial dysfunction. Approximately, 180 genetic mutations of *SOD1* have been identified in patients with ALS (Abel et al., 2012, Farhan et al., 2018) and dominantly inherited mutations link to 15% of fALS cases (Rosen et al., 1993). The pathogenic mechanism is a result of toxic mutant *SOD1* aggregates and oxidative damage observed due to gain-of-function. Mutations like A4V, L84V, H43R, G85R, N86S, G93A have short survival time (SST) and G93C, D90A, H46R have long survival time (LST) for the patients; mutations C146R, A4V, I149T, V148G, D125H mainly induce functional and conformational changes of *SOD1* and others 6 mutations (H46R, H48Q, H48R, H80R, S134N, D125H) reported decreasing the enzymatic activity of the *SOD1* protein.(Srinivasan and Rajasekaran, 2020). *C9ORF72* gene been identified in sALS patients that hexanucleotide (G4C2) repeat expansion is the common cause of ALS(Renton et al., 2011). RNA processing dysregulation appeared to be a key pathway affected in *C9ORF72*-ALS patients (Mejzini et al., 2019). *C9ORF72* mutations resulted in three main disease mechanisms: loss of function of *C9ORF72* protein, a toxic gain of function from sense and antisense *C9ORF72* repeat RNA or DPRs (Balendra and Isaacs, 2018). In 2009, *FUS* was reported as an ALS-related gene that causes both fALS and sALS and is also considered a high-risk gene (Volk et al., 2018). In ALS patients more than 50 *FUS* variants are autosomal dominant. Mutations like R521C and P525L, R521G, R521H, R524W, and G507N have been proposed to give rise to early-onset, antagonistic way lower motor neuron(LMNs) disease with intense neuron damage in the spinal cord and anterior horn along with neuronal and glial cytoplasmic inclusions (Deng et al., 2014). Q290X mutation, which segregates with disease in a large family affected and *FUS* mRNA degraded by nonsense-mediated decay, which results in loss of *FUS* functions (Deng et al., 2014). *TDP-43* is mainly found in the nucleus and plays a crucial role in regulating the splicing mechanism of RNA, providing transcripts stability, biogenesis of miRNAs, cell division, apoptosis and also act as a scaffold for nuclear materials through its interaction with survival motor neuron proteins (SMNs) (Buratti, 2018). Missense mutation like (A315T, M337V, A382T, G348C, and Q343R) presented in C-terminus which is glycine-rich lead to loss of nuclear *TDP-43* and G294V, A328T, S393L have been identified to increase *TDP-43* intrinsic aggregation susceptibility and cause both fALS and sALS (Prasad et al., 2019). *TDP-43* interact with other proteins which disturb by mutations like A315T and M337V and leads to defect in RNA processing mechanisms (Prasad et al., 2019). The mechanism and etiology of the disease are still poorly understood. Omics techniques have provided ways to identify genes and their products related to disease but functional level understanding is still a major challenge. The majority of ALS data present that extensive range of molecular and cellular processes affected by ALS-associated mutations. ALS does not observe due to a single reason or one particular genetic mutation. Based on the fact that in human genes expression and their interaction is under tight temporal. Thus, to gain an understanding of molecular and systems-level network-based analysis of high-throughput data can help to identify diagnostic markers and key candidate genes.

One theory is that some of the ALS-causing proteins are "essential proteins" that, if acted on wrongly by other ALS-causing mutations, would have serious consequences for motor neuron survival. Another possibility is that there are common downstream proteins that interact most effectively with the ALS-causing proteins, either directly or indirectly. The original concept of "essential proteins" was that they are required for survival and that their absence results in lethal phenotype. Traditional methods for finding critical proteins, such as gene knock-out or RNA interference, are frequently time-consuming and expensive. The viability of computational techniques to predict gene essentiality and morbidity has been demonstrated in several previous studies. Proteins rarely perform function individually; instead, they establish a complex network with other genes and proteins to carry out specific biological functions. Protein-protein interaction (PPI) networks are therefore vital for understanding protein functions (Barabasi and Oltvai, 2004), and related diseases (Vidal et al., 2011). Protein-protein interaction (PPI) topological features have been used to identify important proteins in a variety of species (Hahn and Kern, 2005). In a PPIN, nodes of higher degree known as hubs and are more important for biological function as others nodes in the network (Jeong et al., 2001). The key concept is the "centrality-lethality rule," which states that more centralised protein or systems are more lethal (Jeong et al., 2001, Mangangcha et al., 2020). In the PPI network, hubs or bottlenecks, and hubs-bottlenecks linked proteins are more important for survival. Several studies suggest a link between topological centrality (BC) (Pang et al., 2016) and protein essentiality, despite the fact that the centrality lethality rule is still disputed (Batada et al., 2006, Jeong et al., 2001). Systems biology is significantly contributing to biomedical research (Zanzoni et al., 2009). Recent studies have demonstrated drug development techniques for Charcot-Marie-Tooth disease type I (CMT1A) and breast cancer depicting the effectiveness of network pharmacological approaches in discovering medication combinations with high clinical efficacy and minimal side effects (Jaeger and Aloy, 2012). Such system biology approaches are now been applied to Alzheimer’s disease for the identification of novel proteins that are involved in the disease (Soler-Lopez et al., 2012).

A network theory approach to defining connections within complex systems and the generic organising principle of cellular networks are utilised in systems biology. It also aids in the understanding of the function of specific molecules in a variety of cellular processes. Dysregulation of numerous biological processes, cellular component pathways, and molecular activity has a downstream effect such as motor neuron degeneration which has a significant risk associated with ALS (Saez-Atienzar et al., 2021). In our study, we identify, modules that controlled the network from dissortivity and also maintain the flow of information throughout the network of ALS. A set of ALS-associated genes/proteins were used to generate the Amyotrophic Lateral Sclerosis Protein-Protein Interaction Network (ALS-PPIN). We also identified the biological significance, the molecular activity of the modules, and their association with different disease classes. Our interest was to identify the Bn-H and their interaction which may provide an opportunity for a better understanding of ALS.

## 2 Methods

### 2.1 Construction of ALS protein-protein interaction network

**Fig. 1:**
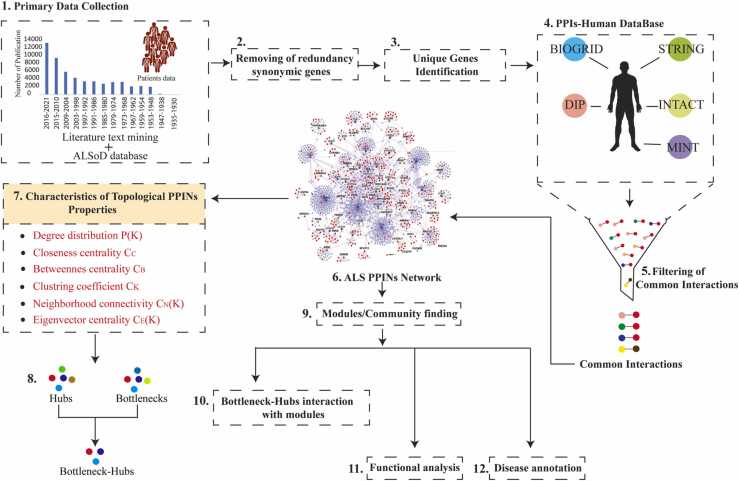
Schematic workflow implemented to study Amyotrophic Lateral Sclerosis protein-protein interaction network.

The genes associated with ALS disease were retrieved from the Amyotrophic Lateral Sclerosis online database (ALSoD) (Abel et al., 2012) and literatures (Chia et al., 2018, Dervishi et al., 2018, Le Gall et al., 2020, Mejzini et al., 2019, Srinivasan and Rajasekaran, 2020). ALSoD database provides comprehensive information about genomic, proteomic, and bioinformatics in association with ALS (Wroe et al., 2008). It also includes patient information like age, clinical data, family history, survival data, sex (Abel et al., 2012). The ALSoD was constructed using genotype, phenotype, and geographical information with associated analysis tools and it transformed from a single gene storage facility recording mutations in the *SOD1* gene to multigene ALS repository ALSoD database links with others databases like ALSgene provides evidence of association to complement the genotype-phenotype association given in ALSoD. It is also used to perform multiple alignment and mutations on *SOD1* gene using ClustalW and jalview to perform multiple sequence alignment in others species for selected genes (Waterhouse et al., 2009). Other links and databases integrated with ALSoD are GeneMANIA used to predict interaction of selected gene (Warde-Farley et al., 2010), a Google Earth API is used to visualise maps of mutations, risk and exposure distributions, HGNC, Enterz Gene, UCSC browser, Protein Structure, OMIM, Genecards, ProtScale, KEGG, Uniprot, iHOP, GeneTest, AmiGO, Ensemble, NCB1, Life Science DB, GeneWiki, WolframAlpha, and WikiGenes. The redundancy in selected genes was removed (duplicate names and aliases names used for the gene). A set of 148 genes were used to generate the Amyotrophic Lateral Sclerosis Protein-Protein Interaction Network (ALS-PPIN) (Fig. 1). These ALS associated genes were used to fetch PPI and to analyse a large number of interactions, we first retrieved the PPIN from five different source databases, namely STRING (von Mering et al., 2003), the General Repository of Interaction Datasets (BioGRID) (Stark et al., 2006), Database of Interacting Proteins (DIP) (Xenarios et al., 2001), IntAct (Hermjakob et al., 2004), Molecular Interactions Database (MINT) (Chatr-aryamontri et al., 2007). We selected those interactions commonly present in at least two of the databases to generate the ALS-PPIN. The constructed network shows a graph denoted by G (*N, E*), where, *N* represents sets of nodes with *N* = ; i = 1,2,., *N* and *E* the sets of edges with *E* = ; i, j = 1,2,3,4,…., *N*.

### 2.2 Statistical analysis of the network

To analyse the topological properties of ALS-PPIN we used various statistical parameters like degree distribution, clustering coefficient, neighborhood connectivity, and centrality parameters (betweenness, closeness, eigenvector) using Cytoscape plugins, Network Analyzer (Assenov et al., 2008), and CytoNCA (Tang et al., 2015).

#### 2.2.1 Degree(k) and probability of degree distributio*n (P(k))*

Degree(k) is a basic characteristic that has an impact on a node’s centrality and is represented by the number of connections a node has to others in a network. The probability of degree distributio*n(P(k*)) is represented by the given equation (Eq. 1).

(1) $$P\left(k\right)=\frac{N_{k}}{N}$$

Where $N_{k}$ represents the total number of nodes with degree k and *N* represents node. The P(*k*) of random and small-world networks follows poison distribution against degree, but for scale-free network, it obeys power-law distribution $P\left(k\right)\sim k^{-\gamma }$ where 4≥γ≤2. The value of γ represent many structural and organizing properties: (i) if γ is2≥γ≤3, several smaller hubs are integrated with few large hub and a large number of separate nodes together, (ii) if γ≤2, consist modules/clusters structure showed that roles of modules are more important, known as hierarchical network, (iii) if γ>3 the hubs are unrelated, losing many scale-free characteristics (Ravasz and Barabasi, 2003).

#### 2.2.2 Clustering coefficient C(*k*)

It indicates the overall organization of formation of clusters in networks and also characterizes the strength of internal connections between the nodes neighborhood in the network. For any a particular node, it is calculated by $C\left(k_{i}\right)=2m_{i}/k_{i}\left(k_{i}-1\right)$ where $m_{i}$ represent the total number of connections with its close neighbors. For scale-free and random networks, C(***k***) ~ constant (Barabasi and Oltvai, 2004) and independent on k and for hierarchical network, $C\left(k\right)∼k^{-\alpha }$ where α∼1 it follows power law-distribution (Borgatti and Everett, 2006).

#### 2.2.3 Neighborhood connectivity $C_{N}\left(k\right)$

Is the number of neighbors connected with a node and it defines the correlation pattern of connectivity for the interacting nodes of the network. $C_{N}\left(k\right)$ can be calculated by (Eq. 2).

(2) $$C_{N}\;\left(k\right)=\underset{q}{\sum }\mathrm{qP}\;(q|k)$$

where $P\left(q|,k\right)$ is the conditional probability of creating a link from a node with a k degree to another node having a q degree(Traag et al., 2013). $C_{N}\left(k\right)\sim \mathrm{constant}$ for scale-free network while for hierarchical network structure $C_{N}\left(k\right)$ follows power law scaling against degree k i.e, $C_{N}\left(k\right)\sim k^{\beta }$, where, β~0.5. If $C_{N}\left(k\right)$ is an increasing function of k, the positive and negative value of exponent *β* showed assortivity or dissortivity nature of the network topology respectively(Virkar and Clauset, 2014).

#### 2.2.4 Betweenness centrality $\left(C_{B}\left(v\right)\right)$

It measures a node occurring several times to bridge along the shortest path between nodes i to j. It is calculated by (Eq. 3).

(3) $$C_{B}\left(v\right)=\sum_{i,j;i\neq j\neq k}\frac{d_{\mathrm{ij}}\left(\nu \right)}{d_{\mathrm{ij}}}$$

where $d_{\mathrm{ij}}\left(v\right)$ denotes the number of geodesic paths connecting node i to node j passing through node *v*. If a node in a network has a high betweenness centrality value, it indicates that the node lies on a path with many other nodes and have the significant ability to propagate information in the network(Borgatti and Everett, 2006).

#### 2.2.5 Closeness centrality $\left(C_{c}\right)$

It is recognised in terms of ‘shortest path lengths’ among the pair of nodes in a network. It can be calculated in terms of farness and is given by (Eq. 4).

(4) $$C_{C}\;\left(k\right)=\frac{N}{\underset{j}{\sum }d_{\mathrm{ij}\;}}$$

where, $d_{\mathrm{ij}}$ is the geodesic distance between pair of nodes *i* and *j*, and N is the nodes present in the network. The higher value of $\left(C_{c}\right)$ for a node means more capability to propagate information in the entire network and the low value represents higher receiving capabilities of information.

Eigenvector centrality ${(C}_{E})$ is proportional to the total sum of the centrality of all neighborhood nodes. It describes the effect of a node on signal processing. It is calculated by (Eq. 5).

(5) $$C_{E}\;\left(i\right)=\frac{1}{\lambda }\underset{j=\mathrm{nn}\left(i\right)}{\sum }v_{j}$$

Where *nn*(i) represents the nearest neighbor of *i* node in the network, with eigenvalue λ and eigenvector $v_{i}$ of eigenvalue equations, $Av_{i}=\lambda v_{i}\;$where A is the network adjacency matrix. $C_{E}$ the function of a node is a powerful indicator of information transmission and depends on the centralities of its neighbors, it varies across different networks(Bonacich, 1987).

### 2.3 Tracing of bottleneck-hubs in the network

In a ALS-PPIN, the nodes with high degree, considered as hubs, are important nodes, because they might be related to disease-causing genes(Barabasi et al., 2011; Lim et al., 2006; Stelzl et al., 2005). The nodes with the high Betweeness Centrality (BC) were defined as bottlenecks. It is believed that these nodes will play an important role in information flow and controlling capability. In the protein-protein interaction network of ALS, we mainly focused to identify the hubs, bottlenecks and bottleneck-hubs (Bn-H) that were the central to the ALS-PPIN. We selected the top 1% highest degree(k) and BC nodes from the overall nodes (1949) present in a network. Further we identified the overlapping of nodes between hubs and bottleneck, which were considered as Bn-H. We also characterise non-Bn-H, non-hub bottlenecks in ALS-PPIN. In order to validate the importance of Bn-H, we also carried out computational knockout experiments. Removal of Bn-H may led to perturbation in the network the phenomenon referred as centrality-lethality rule(Jeong et al., 2001). In our study, to access the network's organisation, change in the absence of the most influential bottlenecks-hubs one at a time, then computed the topological parameters of the modified/reorganized network to assess regulating capacities by evaluating the degree distribution and BC.

### 2.4 Module construction and their interaction with bottleneck-hubs

In the literature, several different types of clustering approaches have been suggested(Huang et al., 2013; Jeong et al., 2001; modeling, 1999; Vidal et al., 2011; Wu et al., 2008). Many frequently used methods are described in terms of comparable data assumptions (e.g., k-means and k-medoids). The k-means (MacQueen, 1967) method has been frequently employed by researchers (Wu et al., 2008) in partitional techniques. The number of groups (k) and a distance measure are required input parameters for this approach. Each data point is first assigned to one of the k clusters based on its distance from the centroids (cluster centres) of each cluster. We constructed the modules/clusters, using the “Molecular Complex Detection” (MCODE) v2.0.0 (Bader and Hogue, 2003), a cytoscape plugin that identify the nodes that are highly interconnected in the form of clusters representing relatively stable, multi-protein complexes that function as a single entity in the network. Clusters in a protein-protein interaction network are commonly protein complexes and pathways, whereas clusters in a protein similarity network are protein families. To separate the dense areas according to provided parameters, the approach uses vertex weighting by local neighbourhood density and outward traversal from a locally dense seed protein. The algorithm has the advantage of having a directed mode, which permits fine-tuning of clusters of interest without considering the rest of the network and analysis of cluster interconnectivity, which is important in protein networks. A known high rate of false positives in data from high-throughput interaction methods has no effect on the algorithm (Bader and Hogue, 2003). The algorithm uses a three-stage process: (i) Weighting: the nodes with the most linked neighbors receive a higher score. (ii) Molecular complex prediction: recursively add nodes to the complex that are over a specified threshold, starting with the highest-weighted node (seed). (iii) Post-processing: filters are applied to increase cluster quality (haircut and fluff). We used defaults values of MCODE, node score cutoff (0.2), haircut, node density cutoff (0.1), K-score (2), maximum depth (100). We selected the top modules based on M-score having values greater than 4. The interaction between the Bn-H and the modules was identified using Cytoscape v3.8.2. which provided the opportunity for a more precise understanding of the biological functions, providing valuable clues for biologists (Nafis et al., 2015).

### 2.5 Gene ontology enrichment and disease association of modules

The five modules were functionally annotated using the gProfiler package(Reimand et al., 2016) to correlate the biological significance. We showed the Gene Ontology classifications like molecular activity, biological process, cellular components of modules using filtering domain size set to “only annotated”, default g:SCS method for multiple testing correction for p-values, maximum p-value set to 0.05, numeric IDs as prefix ENTERZGENE_ACC. Further, we identifying disease-gene correlations for each module because it helps in the understanding of disease mechanisms, which have several applications including disease diagnosis, therapy, and prevention (Luo et al., 2019). The gene-disease class association is identified by the versatile platform “disgenet2r” an R package (Pinero et al., 2020). We used gene2disease function (disgenet2r package) to retrieve the Gene–Disease class association for a given list of genes of each module with search options source database “ALL”, DisGeNET score (0−1). Higher the DisGeNET score shows a stronger correlation between the gene and disease and it showed the Gene-Disease class association in the terms of percentage. Diseases are grouped by their MeSH disease classes, and the Gene-disease association is proportional to the percentage of diseases in each MeSH disease class. The Gene-Disease association score GDAs takes into account the number and type of sources (level of curation, organisms), and the number of publications supporting the association.

## 3 Results

### 3.1 Characterization and statistical analysis of network

**Fig. 2:**
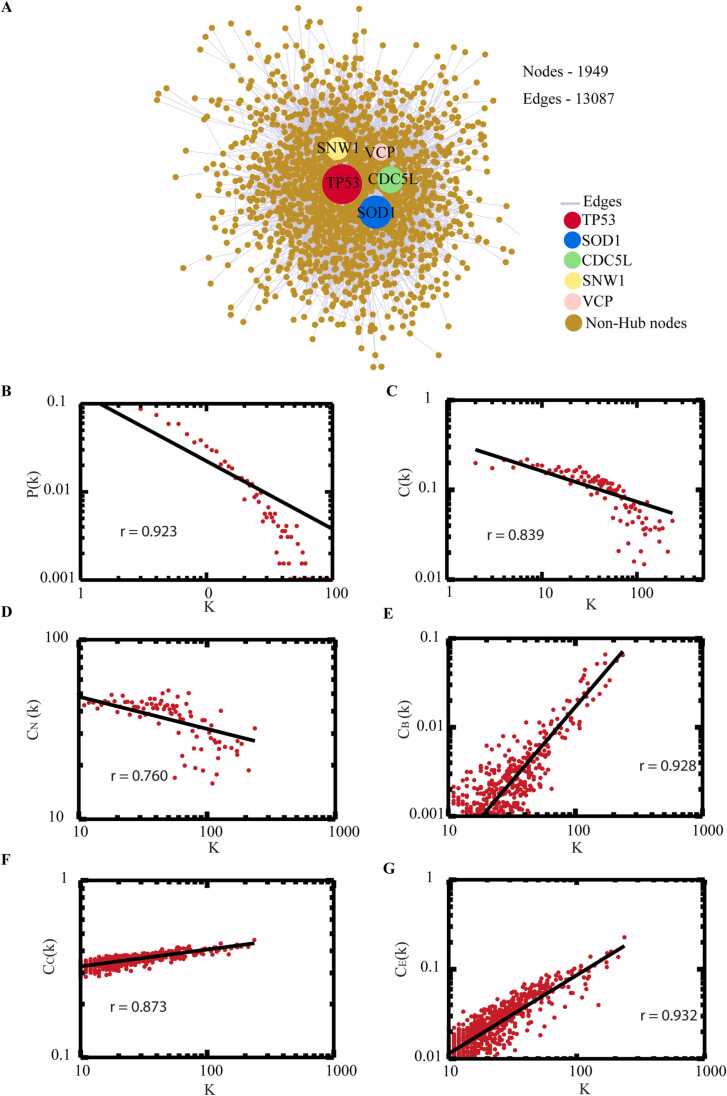
The protein-protein interaction network of ALS and its topological properties. (**A**) The ALS-PPIN is represented in terms of nodes(proteins) and edges (physical interaction). The total number of nodes (1949) and edges (13087) are filled circles (orange) and lines(gray), respectively. The top five significant bottleneck-hubs such as *TP53*(red), *SOD1* (blue), *CDC5L*(green), *SNW1*(yellow), *VCP* (light pink) in the order of size according to a degree, respectively. (**B-G**) The topological and centrality properties of network represented with correlation coefficient values (r) (B) probability of degree distribution *P(k),* (C) average clustering coefficient C(*k*), (D) average neighborhood connectivity (C_N_(k)), (E) betweenness centrality (C_B_), closeness centrality (C_C_), eigenvector centrality (C_E_). All these properties follow the power law scale and show the scale-free hierarchical nature of the network.

We extracted the PPINs commonly present in at-least two of interaction databases out of the five. The ALS-PPIN consists of 1949 nodes and 13087 edges. The results of the topological analysis of degree and BC of all 1949 nodes in (Supplementary Table 1). We retrieved the nodes with top 1% highest degree and BC of total nodes (1949) (Table 1). These nodes considered as hubs and bottlenecks of the network respectively. Out of 19 hubs, 17 hubs were present in bottleneck considered as Bn-H. We also identified that within 17 Bn-H, *CDC5L, SNW1, TP53, SOD1,* and *VCP* were the high degree nodes (hubs) in the ALS-PPIN (Fig. 2**A**). The Bn-H are the most influencing proteins which provide stability and control on the flow of information in the network. The topological analysis of the network depicted that our network followed a power-law distribution against degree. The *(P(k))* obeys power-law distribution $P\left(k\right)\sim k^{-\gamma }$ with a value of exponent γ=0.13±0.76(Fig. 2**B**), where the regression line fitted with the curve to the data point with $P\left(k\right)\sim k^{-0.76}$ with correlation coefficient(r) 0.92 fitted with data. The value $\gamma \left(0.76\right)$ provides hierarchical scale-free behavior to the network including system-level organization consists modules and sparsely distributed Bn-H present in the network (Pastor-Satorras et al., 2001). The clustering coefficient C(*k*) also followed the power-law scale as a function of degree $C\left(k\right)∼k^{-\alpha }$ with negative exponent value of(α=0.35±0.34) which represented that ALS-PPIN follows hierarchical nature. The straight line fitted curve with $C\left(k\right)∼k^{-0.34}$ results a correlation coefficient value $\left(r=0.83\right)$ to the data set (Fig. 2**C**). The Neighbourhood connectivity $C_{N}\left(k\right)$ showed negative exponent value (β=72.87±0.17) given by power law fitting model $C_{N}\left(k\right)\sim k^{\beta }$. The fitted curve line with $C_{N}\left(k\right)\sim k^{-0.17}$ gives correlation coefficient $\left(r=0.76\right)$ and well fitted to the data set points (Fig. 2**D**). The values represented that ALS-PPIN showed a hierarchical scale-free nature along in addition to fractal behaviour due to presence of motifs. All the datapoints of the topological properties fitted power law and verified following a statistical procedure (Clauset et al., 2009). For all the parameters exponential values were calculated using power-law fittings. The network showed a disassortative nature due to the calculated negative value of $\beta_{0}$ the exponent of connectivity parameter and reflects that the Bn-H are still a significant part in regulating the stability of the network. To recognize the importance of the Bn-H nodes strength in signal processing in a network, we used three topological centrality parameters like Closeness centrality${(C}_{c}$), Betweenness centrality${(C}_{B}$) and Eigenvector${(C}_{E}$). In ALS-PPIN these parameters followed power law against degree(K) and showed positive exponents values indicate the strong regulating behaviour of the leading Bn-H. The calculated values of exponents and correlation coefficient(r) of $C_{B}\left(\varepsilon =1.67,r=0.92\right)$, $C_{c}(\eta =0.09,r=0.87,C_{E}(\delta =0.87,r=0.93)$ are respectively. The correlation coefficient value for centrality measure are very high and good fitted with the data (Fig. 2**E-G**). The graph of betweenness against degree showed that high-connecting nodes have more controlling strength to outspread signal throughout the network (Fig. 2**E**). Additionally, the closeness centrality parameter showed that high degree nodes are quickly spread the information as compared to the lower degree nodes which considered as good receiver of propagated signal (Fig. 2**F**). Our network followed a hierarchical scale-free nature means network having modular structure and system level of organization.

**Table 1:** Topological properties of selected 1% highest degree (hubs) and betweeness (bottlenecks) nodes out of total (1949) nodes in ALS-PPIN. The bold highlighted nodes are bottleneck-hubs.

S.NO	Name	Degree (K)	Name	Betweeness (C_B_)
1	**TP53**	236	**VCP**	0.06587026
2	**SOD1**	211	**TP53**	0.06480069
3	**CDC5L**	186	**SOD1**	0.0560067
4	**SNW1**	173	**AR**	0.05264571
5	**VCP**	172	**DISC1**	0.05158762
6	**AR**	171	**ATXN1**	0.04441985
7	**DLD**	155	**SQSTM1**	0.03839771
8	**DISC1**	148	**APP**	0.03540357
9	**NEK4**	138	**CDC5L**	0.03374739
10	**HSP90AA1**	128	**PSEN1**	0.03306112
11	**EGFR**	125	**EGFR**	0.03113805
12	DLST	124	**SNW1**	0.02913541
13	**PDHA1**	122	**HSP90AA1**	0.02680708
14	**ATXN1**	119	**HTT**	0.02414826
15	**HTT**	117	**DLD**	0.02369741
16	**SQSTM1**	117	**PDHA1**	0.02203444
17	**APP**	114	**NEK4**	0.02047508
18	**PSEN1**	110	PARK7	0.02043984
19	EP300	108	MAPT	0.01992754

### 3.2 Tracing of bottleneck-hubs in the network

**Fig. 3:**
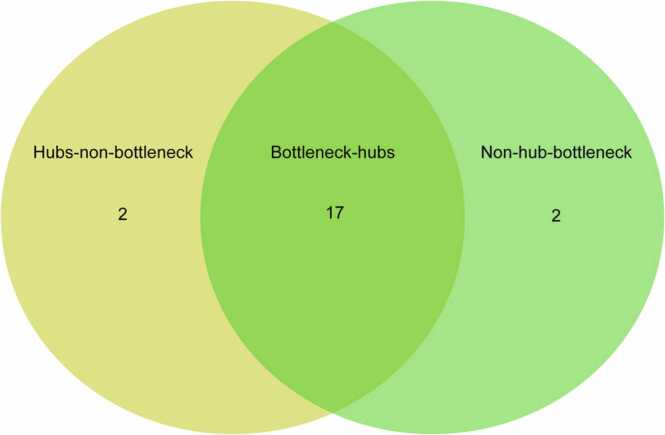
Venn diagram of the number of hubs and bottlenecks.

In the protein-protein interaction network of ALS, we calculated the topological parameters of each node using Network anlayzer (Assenov et al., 2008), the plug-in of Cytoscope v3.8.2 (Tang et al., 2015). The first two parameters, Degree distribution and BC were used to filter the hubs and bottleneck in a network. We selected top 19 proteins (1% of total nodes) in each parameter with highest degree as hubs and BC as bottlenecks. Here, we found 17 nodes are common in both the parameter as Bn-H, 2 non-Bn-H (*DLST* & *EP300*), and 2 non-hub bottlenecks (*PARK7* & *MAPT*) (Fig. 3) (Table 1). Out of 17 bottleneck hubs, we identified the significant hubs, namely *TP53*, *SOD1*, *CDC5L*, *SNW1*, and *VCP* (Fig. 2), with the rationale of high degree nodes (Table 1) as the top five hubs in ALS-PPIN. The node *TP53* had the highest degree (236) followed by *SOD1* (211), *CDC5L* (186), *SNW1* (173) and *VCP* (172). These Bn-H proteins represent as backbone of the network which has great influence on information flow and more control over the network.

In general, important genes may be identified by a series of independent gene knockout experiments. In order to validate the Bn-H, we also carried out computational knockout experiments. Removal of Bn-H may lead to perturbation in the network the phenomenon referred as centrality-lethality rule verified by various genomic investigations (Jeong et al., 2001). The removal of Bn-H does not cause network breakdown, the close interaction of these Bn-H can control network properties and regulatory mechanisms. The accumulation of mutations in any gene causes the dysfunction or removal of expression of a specific protein to become dysfunctional or absent in the cell. In general, this situation re-examining the network's topological properties change may be shown by removing the corresponding hub/hubs (important gene/genes that may have been altered) from the network and then (Peng et al., 2015). If the removal of hub/hubs results in a significant change in network properties, such as breakdown and change in information flow, the network is said to be controlled by the centrality-lethality rule (Jeong et al., 2001). In our study, to access the network's organisation, change in the absence of the most influential Bn-H one at a time, then computed the topological parameter; Degree distribution and BC of the modified/reorganized network to assess the hubs' regulating capacities. The hub removal analysis showed a significant magnitude of changes in network metrics in particular, such as degree and betweenness centrality. In betweenness centrality, all 17 bottleneck-hub were analysed by removing them from the network systematically. We found few Bn-H (*AR*, *SOD1*, *TP53*, *CDC5L*, *VCP*, *EGFR* and *APP*) that allowed significant changes in BC could enhance local and global signal propagation in regulating ALS (Supplementary Fig 1A). The removal of all Bn-H showed a significant control over the interactions of other nodes in the ALS-PPIN. The degree distribution showed mutual control over the regulation of the ALS-PPIN, particularly when we removed *AR* and *SOD1*, whereas the removal of other 15 Bn-H (*TP53*, *CDC5L*, *VCP*, *DISC1*, *SNW1*, *NEK4*, *HSP90AA1*, *EGFR*, *DLD*, *PDHA1*, *ATXN1*, *PSEN1*, *APP*, *SQSTM1*, *HTT*) showed a similar pattern of regulation (Supplementary Fig 1B). In a hierarchical network, hubs, which are not as essential as modules in terms of network control, nevertheless play significant roles in network regulation. Because of the hierarchical nature of the ALS-PPIN, the removal of Bn-H does not cause the network to breakdown. The network's crosstalk among these Bn-H proteins, as well as functional modules, is most likely attempting to maintain the network's structural properties.

### 3.3 Modules of network and their cross-talk with bottleneck-hubs

**Fig. 4:**
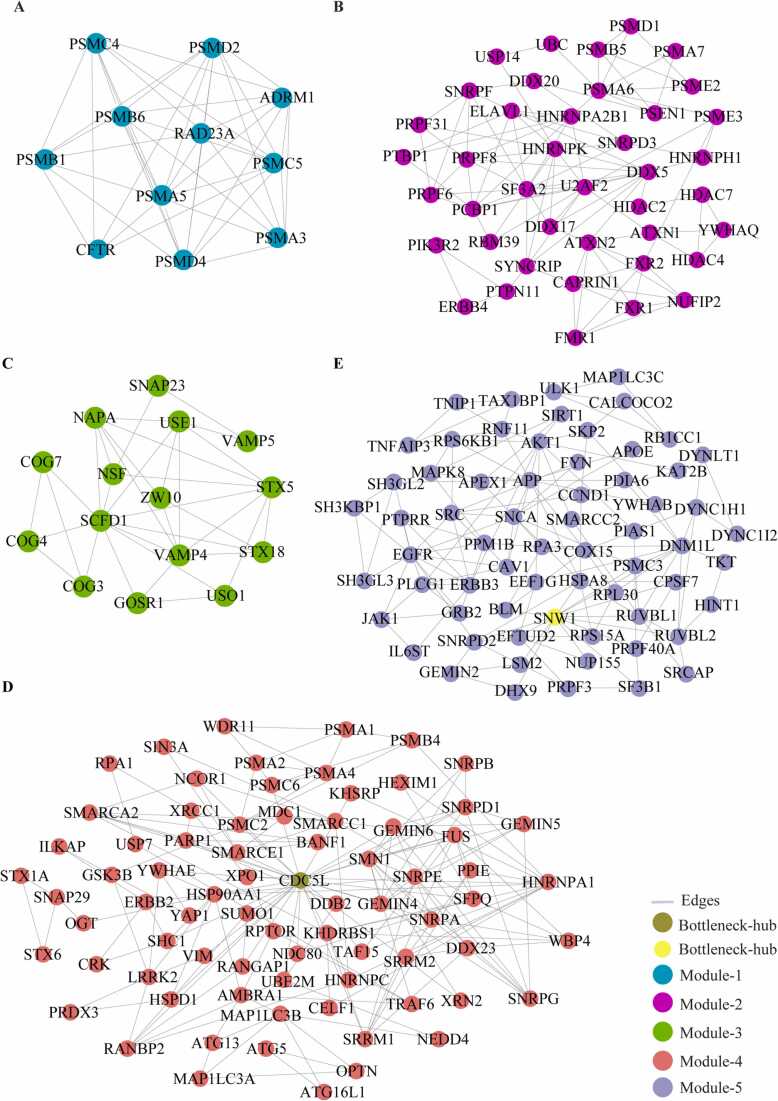
Structure of five modules in PPI network. All the top five modules are constructed and analysed using MCODE. **(A)** All the nodes in module 1 are the filled circle(green cyan) consist nodes(11) edges(44) with scoring value(8.8);**(B)** module 2 are the filled circle(pink) consist nodes(41) edges(103) with scoring value(5.15); **(C)** module 3 are the filled circle(green) consist nodes(15) edges(34) with scoring value(4.85);**(D)** module 4 are the filled circle(red) consist nodes(76) edges(176) with scoring value(4.69););**(E)** module 5 are the filled circle(voilet) consist nodes(65) edges(140) with scoring value(4.37) with the corresponding edges in lines(gray). One of the significant bottleneck-hub (*CDC5L*) present in module 4 as filled circle (dark green) and *SNW1*(yellow) in module 5.

**Fig. 5:**
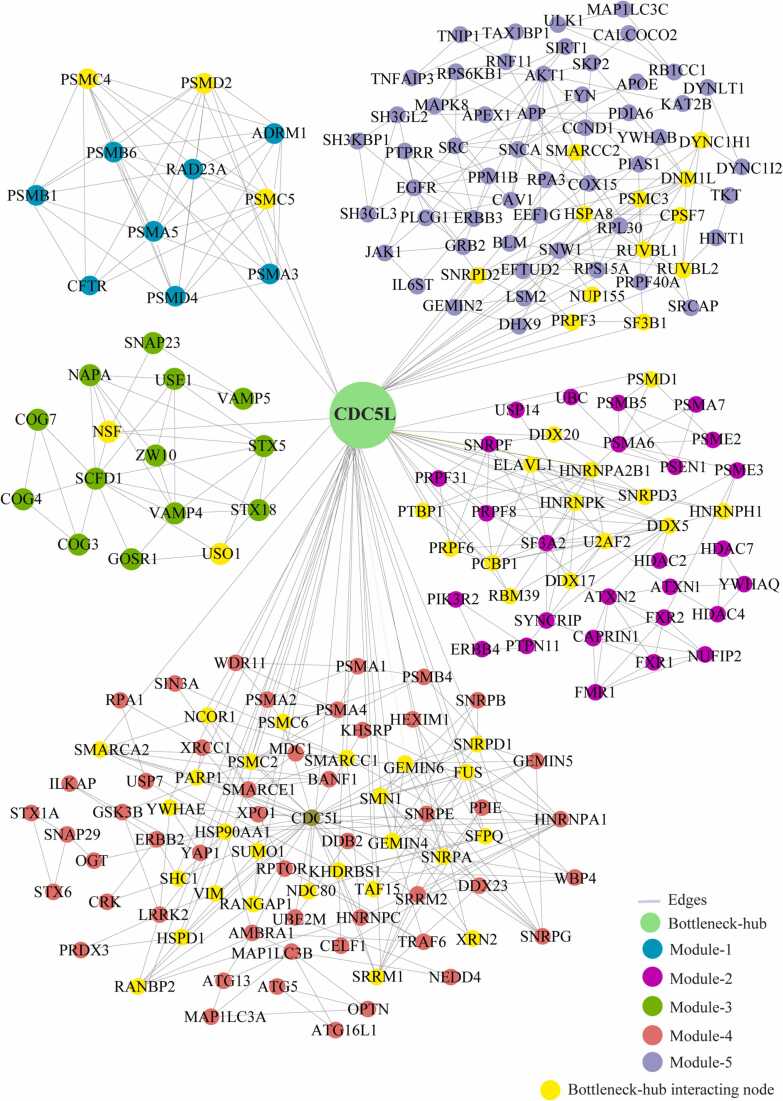
Cross-talk between the significant bottleneck-hub *CDC5L* and five modules in the network. The *CDC5L* bottleneck-hub showed at the centre (green) and its interacting nodes(yellow) of five modules.

**Fig. 6:**
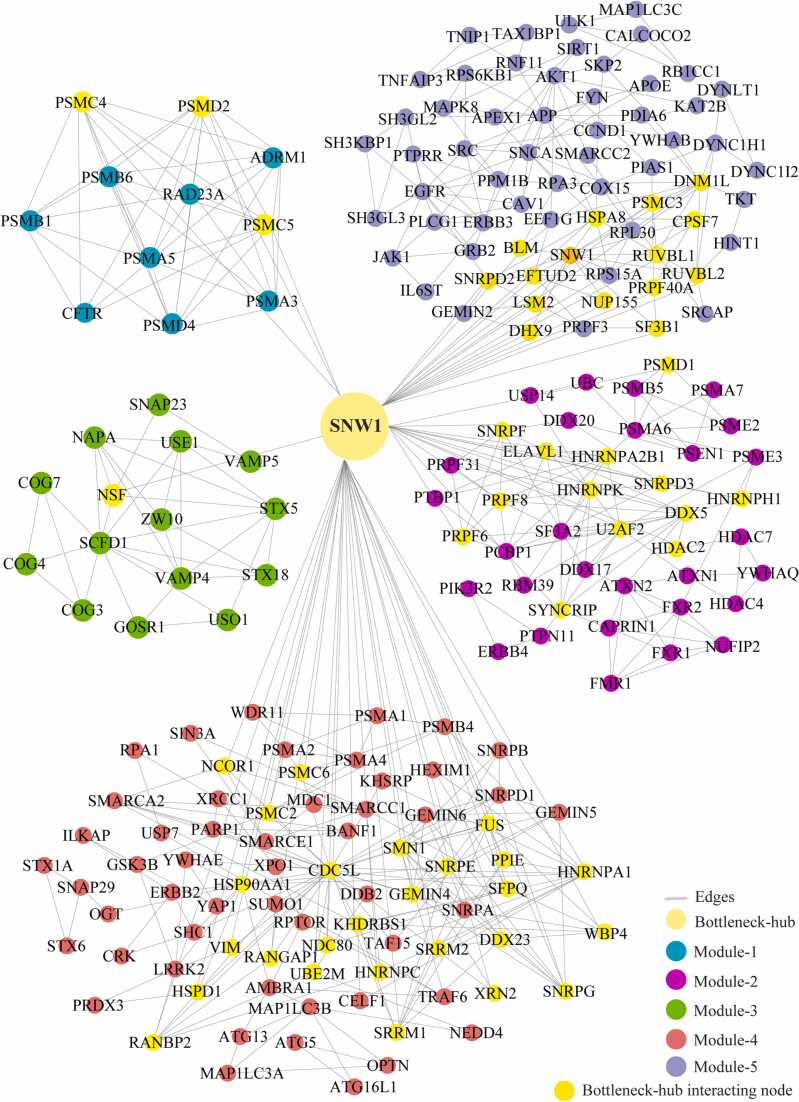
Cross-talk between the significant bottleneck-hub *SNW1* and five modules in the network. The *SNW1* bottleneck-hub showed at the centre (orange) and its interacting nodes(yellow) of five modules.

**Fig. 7:**
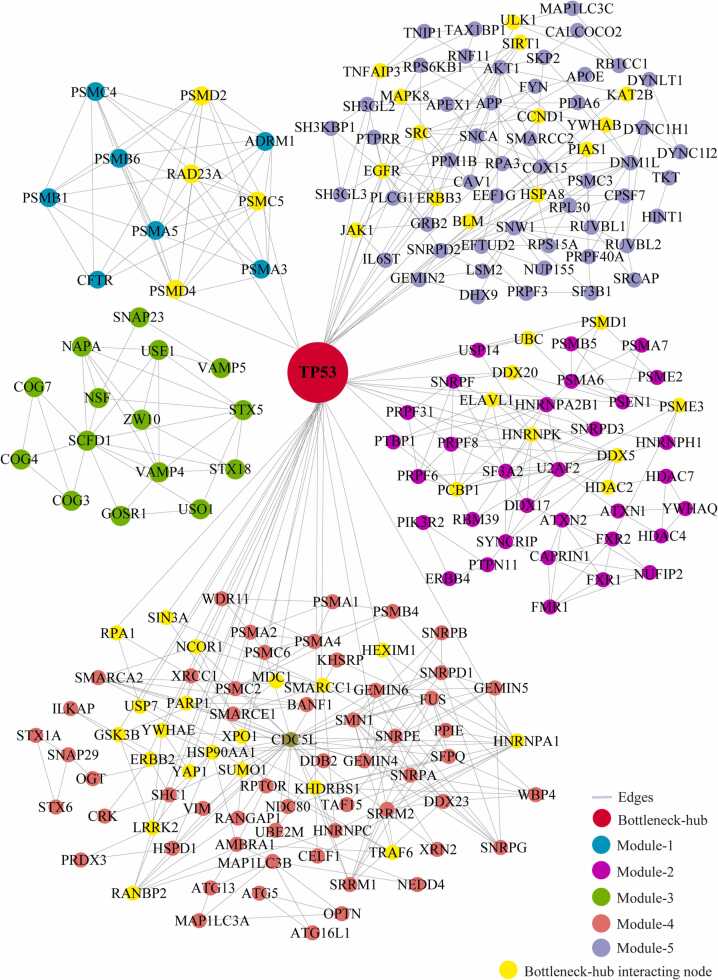
Cross-talk between the significant bottleneck-hub *TP53* and five modules in the network. The *TP53* bottleneck-hub showed at the centre (red) and its interacting nodes(yellow) of five modules.

**Fig. 8:**
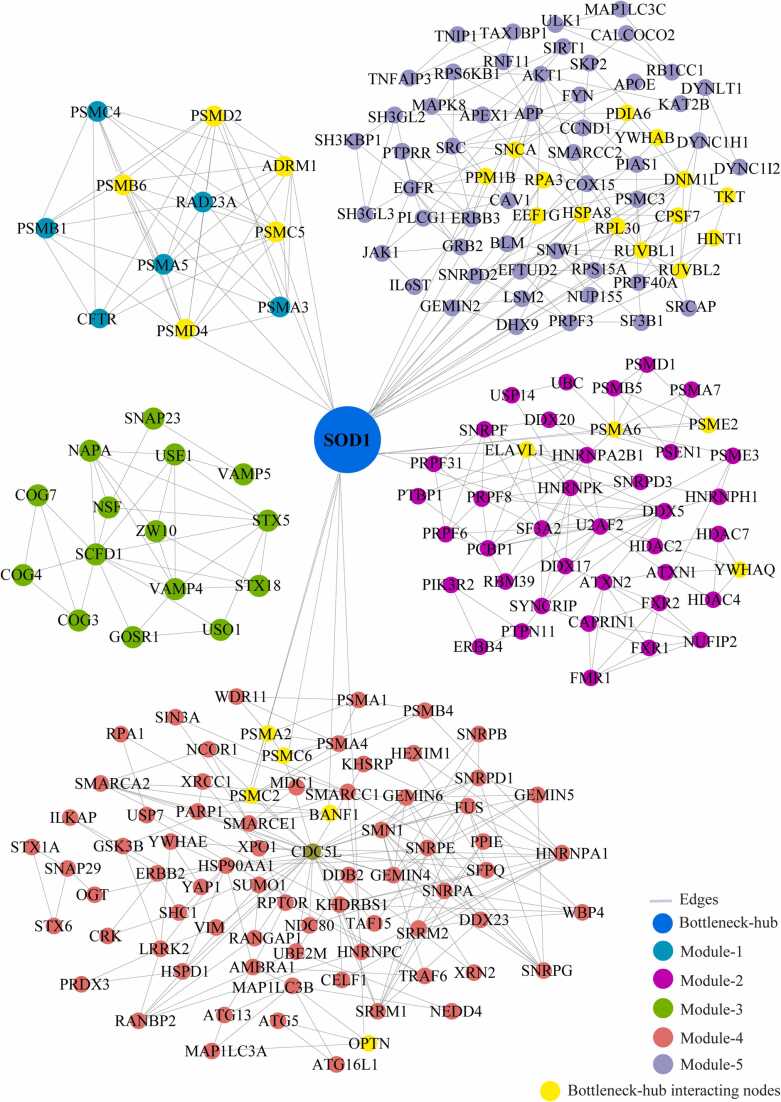
Cross-talk between the significant bottleneck-hub *SOD1* and five modules in the network. The *SOD1* bottleneck-hub showed at the centre (blue) and its interacting nodes(yellow) of five modules.

**Fig. 9:**
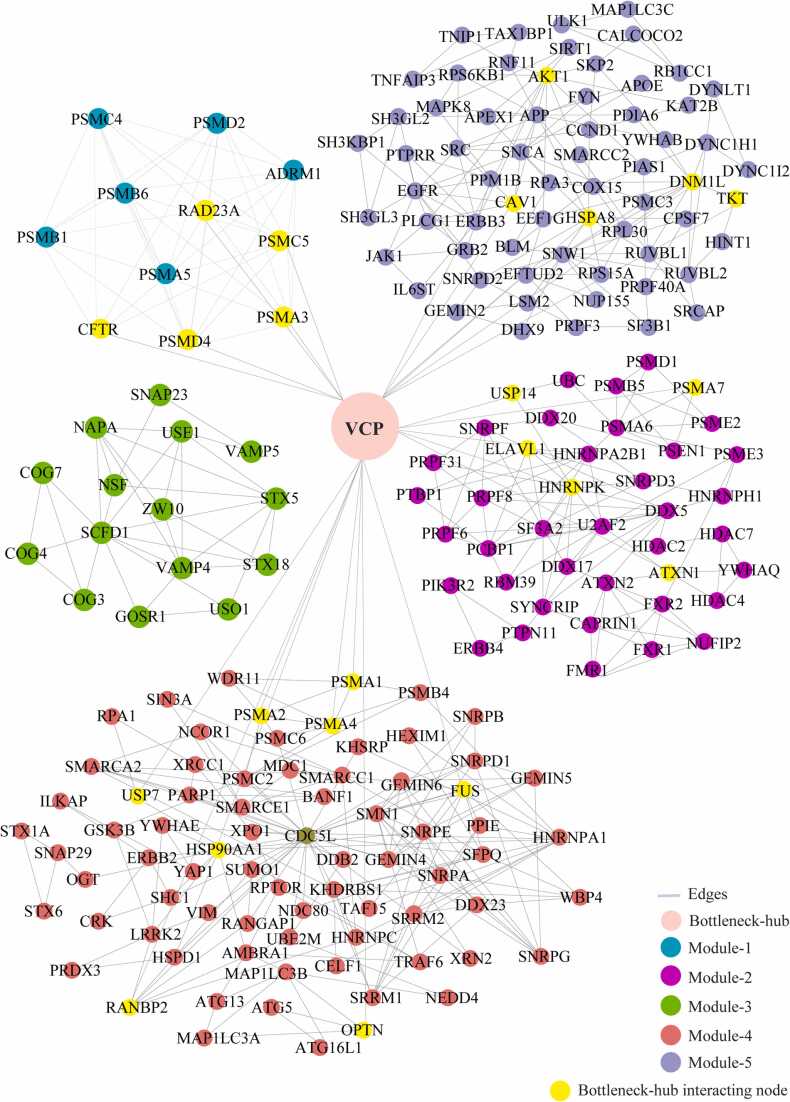
Cross-talk between the significant bottleneck-hub *VCP* and five modules in the network. The *VCP* bottleneck-hub showed at the centre (light pink) and its interacting nodes(yellow) of five modules.

We identified the 29 modules that are highly interconnected regions representing relatively stable, multi-protein complexes that function as a single entity in the network (Supplementary Table 2). We selected the top 5 modules based on the high M-score and the first module had 11 nodes and 44 edges with scoring (8.8). The second, third, fourth, and fifth modules had 41 (5.15), 15 (4.85), 76 (4.69), and 65 (4.37) nodes, respectively, with the corresponding edges of 103, 34, 176, and 140. (Fig. 4) (Table 2). These modules have a system-level organization that was maintained by connecting with other nodes and provide overall functionality to the network (Dong and Horvath, 2007). Out of the five modules, module-4 (*CDC5L)* and module-5 (*SNW1*) consists of one Bn-H, which not only controls the internal regulation of modules respectively; but also affected other modules by interacting with different nodes. Whereas other Bn-H, *SOD1, TP53,* and *VCP* were not observed in any of the five modules suggested that these Bn-H indirectly connected with modular function (Fig. 4). Cross-talk between the modules may be possible due to interaction with common Bn-H and removal of such regulators can affect the functionality of modules and leads to dissortivity of the network. These modules were found to be linked via Bn-H, which have the possibility of cross-talk among the modules. Further we also analysed the interaction of 17 Bn-H with the five modules. Interestingly, we found *CDC5L, SNW1* and *TP53* showed highest strength of interaction with all five functional modules. *CDC5L,* had the largest number of connections with five modules followed by *SNW1, TP53, SOD1, VCP* indicating that these proteins were the key mediators of the modules (Table 3). *CDC5L* and *SNW1* were found to be highly connected with module-4 (28, 26) whereas least with module-3 (2,1) (Fig. 5, Fig. 6). They also showed an interaction with each other in the ALS-PPIN. *TP53, SOD1, and VCP* showed the interactions with modules-1, 2, 4, and 5 with different strength and control the functions of the modules (Fig. 7, Fig. 8, Fig. 9). *SNW1*, *SOD1* and *TP53* had the largest number of connections with a module-5 followed by *CDC5L* and *VCP*. From the analysis of Bn-H and module interaction, we identified that *CDC5L and SNW1* highly regulates one cluster of nodes (module-5, 2 & 4) and *TP53* regulates another cluster of nodes (module-5 & 4). *SOD1* interacted with *VCP* in ALS-PPIN and also showed an interaction with modules-1, 2, 4, and 5. We also identified that *VCP* highly interacted with the module-4 and equally interacted with module-1, 2, and 5. Interestingly we also found among all 17 Bn-H, only *CDC5L*, *SNW1*, *PDHA1* and *PSEN1*showed an interaction with Module-3 (Table-3).

**Table 2:** The number of nodes, edges, and M-code score of top 5 modules of ALS-PPIN.

Modules	M-Score	Nodes	Edges
1	8.8	11	44
2	5.15	41	103
3	4.857	15	34
4	4.693	76	176
5	4.375	65	140

**Table 3:** The botteleneck-hub and their interaction strength with modules. The bold highlighted proteins selected as top bottleneck-hubs serve as the backbone of the ALS-PPIN.

S.NO	Name of bottleneck-hubs	Module 1	Module 2	Module 3	Module 4	Module 5	Total
1	**CDC5L**	3	14	2	28	13	60
2	**SNW1**	3	13	1	26	14	57
3	**TP53**	4	9	0	22	14	49
4	NEK4	0	11	0	16	9	36
5	HSP90AA1	1	3	0	10	15	29
6	**SOD1**	5	4	0	5	14	28
7	EGFR	1	5	0	6	13	25
8	DLD	4	5	0	8	8	25
9	**VCP**	5	5	0	8	5	23
10	AR	0	4	0	6	12	22
11	PDHA1	0	3	2	5	6	16
12	ATXN1	1	4	0	2	7	14
13	PSEN1	3	4	1	5	5	18
14	DISC1	0	1	0	6	4	11
15	APP	0	3	0	5	12	20
16	SQSTM1	1	3	0	11	15	20
17	HTT	4	5	0	10	13	32

### 3.4 Functional enrichment analysis of modules

**Fig. 10:**
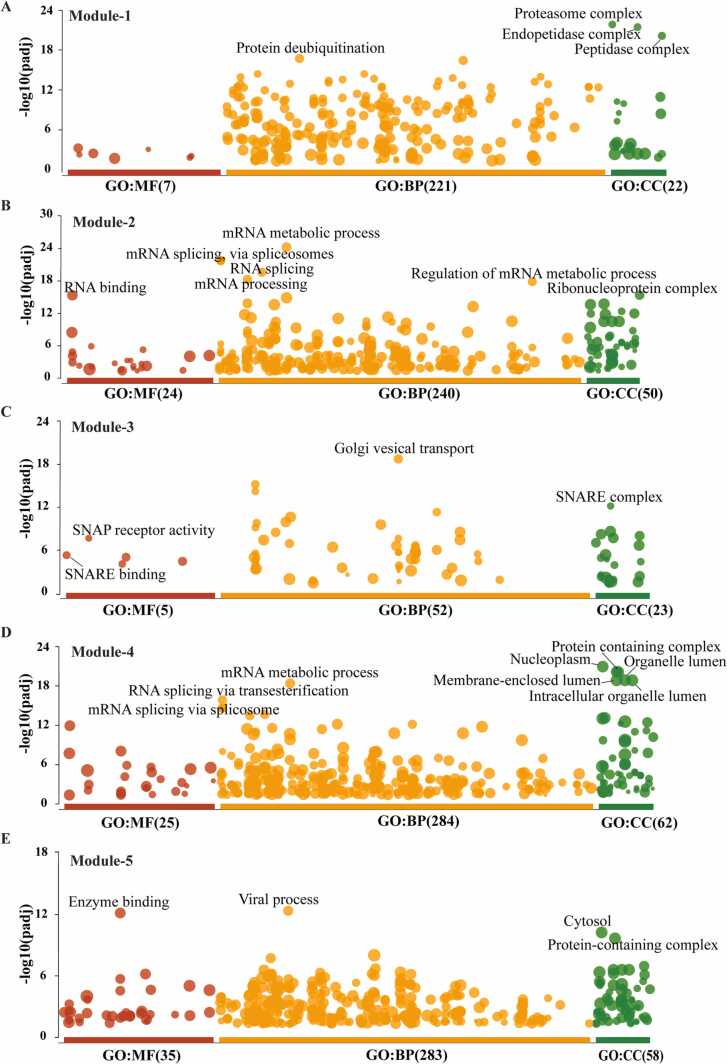
Manhattan plot that represents the enrichment analysis of five modules. The x-axis represented functional term’s Molecular function (GO: MF) is red; Biological process (GO: BP) is orange and Cellular component (GO: CC) is green. The sizes of the filled circle according to the term size, means larger terms have larger circles. The y-axis shows the adjusted enrichment p-values in the negative log10 scale.

We performed the functional enrichment analysis for all five modules. Module-1 was enriched with proteins having proteasome-activating activity, endopeptidase activity and are associated with biological processes such as protein deubiquitination, protein modification, proteasome protein catabolic process. These proteins were found to be localized in proteasome complex, endopeptidase complex, peptidase complex (Fig. 10**A**). The Module-2 proteins were found to be enriched in RNA binding function and regulate mRNA processing, mRNA splicing via spliceosome, mRNA metabolic process. These proteins were found mainly in the ribonucleoprotein complex (Fig. 10**B**). The proteins of module-3 were present in the SNARE complex and are involved in Golgi vesical transport process by binding to the SNARE complex. These proteins were also involved in SNAP receptor activity (Fig. 10**C**). Module-4 showed the involvement of the protein in binding to various macromolecules such as an enzyme, nucleic acid, RNA, DNA that perform RNA splicing via transesterification, mRNA splicing via spliceosome, and are mainly found in nucleoplasm, membrane-bound lumen, intracellular organelle lumen (Fig. 10**D**). Proteins associated with module-5 are mainly present cytosol, protein-containing complex, nucleus, membrane enclosed lumen performing molecular functions such as enzyme binding, protein binding, kinase binding thus regulating the viral process, cell cycle, protein modification process (Fig. 10**E**). We also analysed that the *CDC5L* present in module-4, and from the result of functional enrichment it was related to mRNA metabolic process, suggesting that the influence of the *CDC5L* in mRNA metabolism using spliceosome and transesterification activities. Furthermore, the *SNW1* also present in module-5, which is functionally associated with enzyme binding as molecular function and also involved in viral process in the cytoplasm.

### 3.5 Module-disease class associations

The module-disease association is identified by DiSGenet tool of each module (module1–5). We found that the proteins of all five modules were associated with different disease classes like neoplasm, nervous system disorder, digestive, respiratory tract, cardiovascular, mental disorder, immune system, hemic and lymphatic, congenital, hereditary, neonatal, and others. Disease-associated with these modules depicts the majority of genes of module-1 showed association with neoplasm disease (40%), immune system disease (30%), and nervous system disease (30%). *PSMC4* gene present in module-1 showed high association with neoplasm (40%) as well as nervous system (30%) while it does not show association with immune system disease (Supplementary Fig. 2). In module-2 proteins are highly associated with neoplasm (50%), pathological conditions (40–50%), nervous system (30%), cardiovascular disease (40%), and digestive system disease (20%). *SNRPD3* and *U2AF2* showed (50%) the highest association; *DDX5, DDX17,* and *SNRPF* in between (40–50%) with neoplasm class. *SN3A2* the proteins showed 50% association with the pathological condition and cardiovascular disease*. ATXN1, ATXN2, FMR1, FXR2, NUFIP2, PRPF6, PRPF8, PSEN1* were mostly associated with nervous system disorder (30–40%) (Supplementary Fig. 3). Module 3 proteins were highly associated with neoplasm disease (40–50%), nervous system disease (60%), congenital, hereditary, and neonatal disease and abnormalities (20–30%) while these proteins were least associated with chemically induced disorders (1%), otorhinolaryngologic disease (<10%) and male urogenital disease (<10%). Among genes of module-3, *NAPA* showed the strongest association (60%), *SCFD1,* and *STX5*(30–40%) suggesting their possible role in the etiology of nervous system diseases (Supplementary Fig. 4). Among all the modules, module-4 showed the highest association with different disease classes. The majority of proteins are associated with neoplasm disease, pathological conditions, nervous system diseases, digestive system disease. These proteins have a negligible association with occupational disease, chemically-induced disorders. *HSPD1* and *PSMB4* were observed to show strongly interrelated with neoplasm disease (80%) while *GEMIN6* is highly interrelated with nervous system diseases (80%). *SNRPG* showed 50% association with a pathological condition, skin, and connective tissue disease class (Supplementary Fig. 5). Furthermore, the major group of proteins in module-5 were strongly linked to neoplasms (60%), nervous system disease (40%), pathological conditions (30%). Several proteins like *CPSF7, EEF1G, RNF11, RPA3, RPL30, SH3GL3, SKP2, SNW1* were highly correlated with the neoplasm’s disease (50–60%). The nervous system disease class consists of proteins *APP, DYNC1H1, DYNC1I2, HINT1, LSM2, RNF11, SNCA* in between 30% and 40% association. One protein *DYNLT1* showed a major association with respiratory tract (60%), cardiovascular and male urogenital (30–40%) (Supplementary Fig. 6).

## 4 Discussion

In this study, we applied a computational systems biology approach to investigate the ALS-PPIN. To construct the ALS-PPIN, we used five different human databases STRING, BIOGRID, DIP, IntACT, and MINT. Finally, we constructed ALS-PPIN consist of 1949 nodes and 13087 edges. It followed a hierarchical scale-free behaviour. The nodes with number of links that greatly exceeds the average value were considered as hubs (Chen et al., 2019, Pang et al., 2016). In the ALS-PPIN, we considered high-degree nodes as hubs. In order to filter the potential hub, we selected 19 highest degree nodes (1% of total nodes (1949)) which could regulate the ALS-PPIN. Further, we also selected 19 highest BC (1% of the total nodes) in the network, considered as bottleneck. They can characterize information flow and predict the most influencing candidate in the network.

Among the 19 hubs, we found only 17 hubs were also present in selected 1% of BC nodes which were considered as Bn-H, could provides the stability and control the information flow in the network (Table 1). Further, out of 17 bottleneck hubs, we identified the significant hubs, namely *TP53*, *SOD1*, *CDC5L*, *SNW1*, and *VCP* with the rationale of high degree nodes in the ALS-PPIN (Fig. 2). *CDC5L*, *TP53*, *SNW1, SOD1, VCP* were five top hubs as well as Bn-H which were the dominant preservers of the topological properties of the network. Interestingly, we identified *SOD1* as a Bn-H which is already reported as a high-risk gene in ALS (Rosen et al., 1993). *CDC5L*, *TP53*, *SNW1, SOD1,* and *VCP* have significant roles because they interact with functional modules with high number of strengths as compared to other Bn-H. The correlation between Bn-H and modules gave an idea that Bn-H also controlled the regulation of these modules. Based on Bn-H and module interaction we found *CDC5L*, had the largest number of connections with five modules followed by *SNW1* and *TP53*; indicating that these proteins were the key mediators of the modules (Table 3). Bn-H could preserve the stability of the network, information was fast and molecules were quickly accessible by directly interacting with the nodes in the module (Nafis et al., 2015). Out of 17 significant Bn-H, only *CDC5L* was present in module 4 and *SNW1* in module 5, which was the influencing node with strong cross-talking among the module that interacted with it. *SOD1*, *TP53,* and *VCP* were not present in any of the five modules, acted as mediators to cross-talk among the modules and also indirectly interfered with modular properties and activities. Because these Bn-H are strongly sensitive in preserving the topological properties of the network, their absence causes the breakdown of the network. Therefore, acting as the key mediator in the regulation of ALS-PPIN. Modules that perform significant functions in the network were constructed. In this study, the correlation between Bn-H and modules interaction gave an idea that Bn-H were also responsible in controlling the regulation of these modules.

In module-1, *CDC5L* interacts with three proteins *PSMC4, PSMD2,* and *PSMC5* mainly participate in protein homeostasis and cell processes like DNA damage, apoptosis, cell cycle progression. In module-2, five proteins (*PSMD1, DDX5, PCBP1, ELAVL1,* and *HNRNPK)* were interacting with both *CDC5L* and *TP53* which are functionally enriched with mRNA splicing mechanism which is known to be highly affected in ALS (Butti and Patten, 2018). Interestingly in module-4, we found *CDC5L* connected with *FUS* protein, which is one of the high-risk proteins of both familial and sporadic type ALS (Volk et al., 2018), Dementia (Mompean and Laurents, 2017), and Parkinson’s disease (Yan et al., 2010). *TAF15* and *FUS* affect the turnover of their RNA targets in ALS (Kapeli et al., 2016). The interaction of *CDC5L* with module-5 proteins which were functionally associated with enzyme activity, neoplasm, and nervous system diseases.

In module-1, *SNW1* showed interaction with three proteins (*PSMC5, PSMC4, PSMD2*) involved in protein metabolism and other cellular function like DNA damage, cell cycle progression. Thirteen proteins showed interaction with *SNW1*in module-2 (Fig. 6) which are functionally enriched with mRNA processing process. *SNW1* also showed the interaction with *NSF* in module-3, which is functionally associated with SNARE binding. *NSF* protein functionally important for the delivery of cargo proteins to all compartment of the Golgi stack and highly associated with neoplasm and nervous system diseases**.** In module-4, *SNW1* interacting with more than 25 proteins which are functionally enriched with mRNA processing. *SNW1*, have been associated with cell migration in glioblastoma (Ramos et al., 2019) and also reported in axonal transport implication during tau organization related to Alzheimer’s disease(Ivashko-Pachima and Gozes, 2019). Interestingly, *SNW1* shown direct interaction with *CDC5L* (157–536 residue), it functions as a coactivator that may couple vitamin D receptor-mediated transcription and RNA splicing (Zhang et al., 2003). In module-5, *SNW1* showed interaction with 14 proteins involved functionally in enzyme activity mostly observed in ALS disease progression (Srinivasan and Rajasekaran, 2020). Two proteins of module-5, *LSM2* (also known as snRNP) and *HSPA8* both are reported as ALS causative proteins. Mutation in *FUS/TLS* protein reduced gems formation, altered snRNPs in patients’ fibroblasts and transgenic mice. Along these lines, it is also observed that *FUS/TLS* mutations reduce interaction with U1-snRNP (Sun et al., 2015, Yu et al., 2015). Post transcriptional inhibition of *HSPA8* expression leads to synaptic vesicle cycling defects in multiple models of ALS (Coyne et al., 2017).

*TP53* showed interaction with (*PSMD4, PSMD2, PSMC5, RAD23)* of module-1 involved in the protein deubiquitination process. In modules-2, the interaction of *TP53* with *HDAC2, DDX5, PCBP1, PSME3, ELAVL1, DDX20, PSMD1, UBC, HNRNPK* were enriched in mRNA splicing. The dysregulation or inhibition of protein deubiquitination (Cykowski et al., 2019) and mRNA splicing (Butti and Patten, 2018) processes favor ALS. Phosphorylation of *HNRNPK* by cyclin-dependent kinase 2 controls the cytosolic accumulation of *TDP-43* in ALS (Moujalled et al., 2015). Interestingly, *HNRNPK* and *DDX5* proteins are transcriptional coactivators of *TP53* and help to regulate the intrinsic apoptotic pathway in response to the DNA damage process observed in ALS (Pelisch et al., 2012). *DDX20* is associated with a neurogenerative disease SMA (Charroux et al., 1999). Functional loss of *DDX20* was observed in ALS on disruption of high-risk gene *FUS* (Cacciottolo et al., 2019)*.* In modules-4, *TP53* interacting proteins are majorly enriched with mRNA splicing function and highly associated with neoplasms diseases. *SMN1* interacted with *TP53* in module 4 reported in SMA (Singh and Singh, 2019), and ALS (Chi et al., 2018). *TP53* showed interaction with modules − 5 proteins which are functionally enriched with enzyme activity that altered process in ALS(Srinivasan and Rajasekaran, 2020).

Association of *SOD1* with module-1 proteins (*PSMD2, ADRM1, RAD23A)* were involved in ALS15-type with or without Frontotemporal dementia (Huttlin et al., 2017). The proteins of module-2 (*PSME2, PSMA6, ELAVL1, YWHAQ)* and module-4 (*OPTN, PSMC2, PSMA2, PSMC6, BANF1*) are enriched with RNA processing and protein homeostasis whose disrupts function observed in ALS (Butti and Patten, 2018, Mejzini et al., 2019). Interestingly, *SOD1* showed interaction with *OPTN* involved in neuroinflammation, autophagy, and vesicular trafficking in ALS (Maruyama et al., 2010), FTD (Hortobagyi et al., 2011), Alzheimer’s disease, and Huntington’s disease (Schwab et al., 2012). *SOD1* showed more interaction with module-5 which is highly associated with enzymatic activity. The proteins (*SNCA, HSPA8, HINT1*) are associated with neurodegenerative diseases like Parkinson’s, Alzheimer’s, Huntington's, Prion disorders, and Fronto-temporal dementia (Lin et al., 2020, Matsumura et al., 2013, Shchagina et al., 2020, Sung et al., 2005, Wyttenbach and Arrigo, 2009). The nodes in module-5 (*RUVBL1, RUVBL2)* interacts with *HINT1* and modulates *TP53* levels and *TP53*-mediated apoptosis (Weiske and Huber, 2006).

Many studied reported that more than 50 missense mutations in gene coding *VCP* is causative in many neurodegenerative diseases characterised by ALS, FTD, IBM, CMT2Y, and PBD (Al-Tahan et al., 2018). Approximately 9% of patients with *VCP* mutations had ALS phenotype, 4% with Parkinson's disease, and 2% has been diagnosed with Alzheimer's (Al-Obeidi et al., 2018). In module-1, *VCP* interacts with 5 proteins which are functionally associated with protein metabolism and mostly associated with neoplasms disorders. *VCP* showed interaction with (*ELAVL1, HNRNPK, ATXN1, PSMA7, USP14*) of module-2 play important role in protein homeostasis process which is mostly affect in ALS. A functional deficiency of *VCP* observed contributes to impaired DNA repair in multiple polyglutamine diseases. Although normal and mutant polyglutamine proteins (*ATXN1*) interact with *VCP*, only mutant protein affect dynamism of *VCP* (Fujita et al., 2013). In module-3 *VCP* showed no interaction with any of the proteins. In module-4 *VCP* interacts with 8 proteins (*HSP90AA1, USP7, PSMA1, PSMA2, PSMA4, FUS, RANBP2, OPTN*) functionally enrichment with mRNA processing. Both *FUS* and *OPTN* are well known studied protein to cause ALS and cause aberrant protein homeostasis due to various mutations. *FUS* protein reported as one of the top 4 high risk genes to cause both sALS and fALS (Volk et al., 2018). Q290X mutation leads to *FUS* mRNA degraded by nonsense-mediated decay, which results in loss of *FUS* functions and cause ALS (Deng et al., 2014). *OPTN* is an autophagy receptor and mutations in the *OPTN* gene result in familial glaucoma (E50K) and ALS (E478G) reportedly abolishes its NF-κB suppressive activity (Nakazawa et al., 2016, Shen et al., 2015). In module-5, *VCP* shown interaction with 5 proteins (*AKTI, CAV1, HSPA8, DNM1L, TKT)* functionally associated with enzyme binding processes and most of these genes are mostly related to neoplasms disorder followed by nervous system diseases. role of *DNM1L* seen in abnormal mitochondrial dynamics, mitochondrial fragmentation, autophagy/mitophagy, and neuronal damage in alzheimer’s disease and other neurological diseases, including Parkinson's, Huntington's, ALS, multiple sclerosis, diabetes, and obesity (Oliver and Reddy, 2019, Vantaggiato et al., 2019). Analysis reveals that *LanCL1* is a positive regulator of *AKT1* activity, and *LanCL1* overexpression restores the impaired *AKT1* activity in ALS model mice (Tan et al., 2020). Neuron-targeted *CAV1* improves neuromuscular function and extends survival in SOD1^G93A^ transgenic mice (Sawada et al., 2019).

We also found 29 node/protein, showed a common connection between the five Bn-H (*CDC5L, SNW1, TP53, SOD1, and VCP*) and others also in ALS-PPIN. *HSPA5* nodes showed interaction with 10 Bn-H (*APP, AR, ATXN1, CDC5L, EGFR, HSP90AA1, PSEN1, SNW1, SQSTM1,* and *TP53*) whereas *HSPA8* with (*APP, ATXN1, CDC5L, HSP90AA1, HTT, PSEN1, SNW1, SOD1, TP53, and VCP*). *HSPA5* majorly associated with digestive system disorders and neoplasm (Liver carcinoma) (Feng et al., 2019, Shu et al., 2020). *HSPA5* (*GRP78*) activates the Wnt/HOXB9 pathway to promote invasion and metastasis of hepatocellular carcinoma by chaperoning *LRP6* (Xiong et al., 2019*)*. Post transcriptional inhibition of *HSPA8* expression leads to synaptic vesicle cycling defects in multiple models of ALS (Coyne et al., 2017). *HSPA8* mostly reported in many neurodegenerative diseases like Parkinson’s, Alzheimer’s, Huntington's, Prion disorders, and Fronto-temporal dementia (Lin et al., 2020, Matsumura et al., 2013, Sung et al., 2005). Whereas, *GAPDH* is another node showed interactions with 9 Bn-H (*APP, AR, ATXN1, CDC5L, HSP90AA1, HTT, PSEN1, SOD1, TP53*). The alteration in *GAPDH* function is associated with oxidative stress in ALS (Pierce et al., 2008). S-nitrosylated *GAPDH* mediates neuronal apoptosis induced by ALS-associated mutant SOD1^G93A^ (Lee et al., 2016). *GAPDH* expression defects were also found in muscles from ALS patients (Desseille et al., 2017). The nodes (*PARK7, HSPA4, CCAR2*) were found as an intermediator for *SOD1, TP53, AR* and *HSP90AB1, RUVBL1* for *AR, SOD1, CDC5L*. In future, biochemical investigation of the observed Bn-H and their interacting partners could provide further understanding to prioritize key genes and their role in the pathophysiology of ALS.

## Ethics declarations

Not applicable.

## CRediT authorship contribution statement

**Rupesh Kumar:** Conceptualization, Data curation, Investigation, Writing – original draft. **Shazia Haider:** Conceptualization, Writing – review & editing, Supervision.

## Conflicts of Interest

The authors declare that they have no conflict of interest.

## Appendix A Supplementary material

**Supplementary Table 1:** The values of all the six topological properties of all the nodes (1949) forming ALS-PPIN.

.

**Supplementary Figure 1:** The representation of the network properties, such Degree distribution (Pk), and Betweenness centrality (BC) of removal of bottleneck-hubs in the ALS-PPIN.

.

**Supplementary Figure 2:** Representation of modules-1 genes association with the different disease classes.

.

**Supplementary Figure 3:** Representation of modules-2 genes association with the different disease classes.

.

**Supplementary Figure 4:** Representation of modules-3 genes association with the different disease classes.

.

**Supplementary Figure 5:** Representation of modules-4 genes association with the different disease classes.

.

**Supplementary Figure 6:** Representation of modules-5 genes association with the different disease classes.
