# Supplementary material for: Protein network analysis to prioritize key genes in amyotrophic lateral sclerosis
Source: IBRO Neurosci Rep. 2021 Dec 7;12:25–44. doi: 10.1016/j.ibneur.2021.12.002 (PMC8669318; doi:10.1016/j.ibneur.2021.12.002)
Supplement: Supplementary file 2 — Supplementary material. [file mmc2.docx]

| **Modules** | **M-Score** | **Nodes** | **Edges** |
| --- | --- | --- | --- |
| 1 | 8.8 | 11 | 44 |
| 2 | 5.15 | 41 | 103 |
| 3 | 4.857 | 15 | 34 |
| 4 | 4.693 | 76 | 176 |
| 5 | 4.375 | 65 | 140 |
| 6 | 4 | 4 | 6 |
| 7 | 4 | 4 | 6 |
| 8 | 3.5 | 45 | 77 |
| 9 | 3.333 | 4 | 5 |
| 10 | 3.333 | 4 | 5 |
| 11 | 3.25 | 17 | 26 |
| 12 | 3.2 | 11 | 16 |
| 13 | 3.077 | 14 | 20 |
| 14 | 3 | 3 | 3 |
| 15 | 3 | 3 | 3 |
| 16 | 3 | 3 | 3 |
| 17 | 3 | 3 | 3 |
| 18 | 3 | 3 | 3 |
| 19 | 3 | 3 | 3 |
| 20 | 3 | 9 | 12 |
| 21 | 3 | 3 | 3 |
| 22 | 3 | 3 | 3 |
| 23 | 2.867 | 31 | 43 |
| 24 | 2.778 | 19 | 25 |
| 25 | 2.727 | 12 | 15 |
| 26 | 2.667 | 4 | 4 |
| 27 | 2.667 | 4 | 4 |
| 28 | 2.593 | 28 | 35 |
| 29 | 2.5 | 5 | 5 |

**Supplementary Table 2**: Number of nodes, edges and M-code score of all 29 modules of ALS-PPIN.
