# Supplementary figures and images for: Protein network analysis to prioritize key genes in amyotrophic lateral sclerosis
Source: IBRO Neurosci Rep. 2021 Dec 7;12:25–44. doi: 10.1016/j.ibneur.2021.12.002 (PMC8669318; doi:10.1016/j.ibneur.2021.12.002)

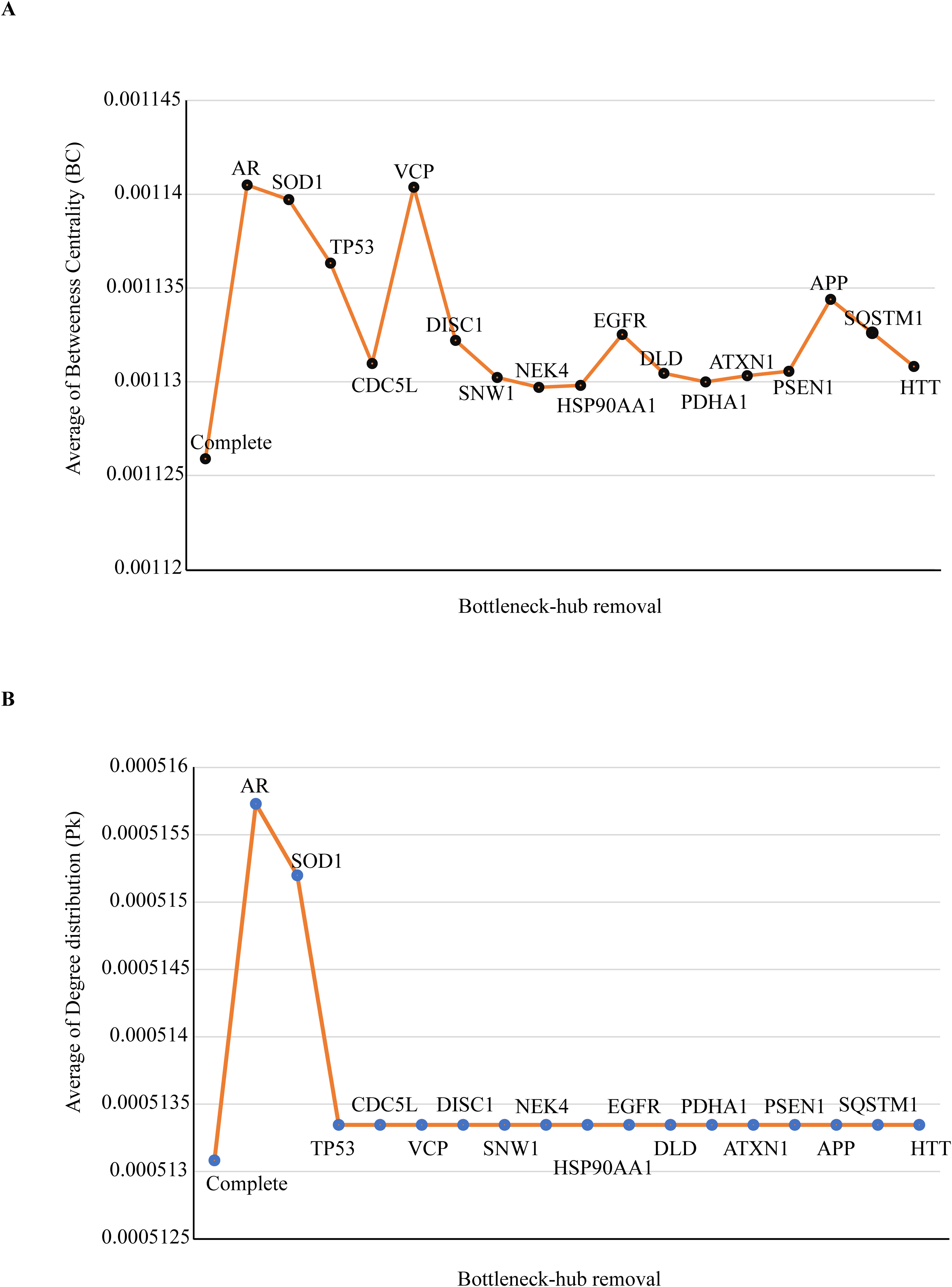

Supplement: Supplementary file 3 — Supplementary Figure 1: The representation of the network properties, such Degree distribution (Pk), and Betweenness centrality (BC) of removal of bottleneck-hubs in the ALS-PPIN. [file mmc3.jpg]

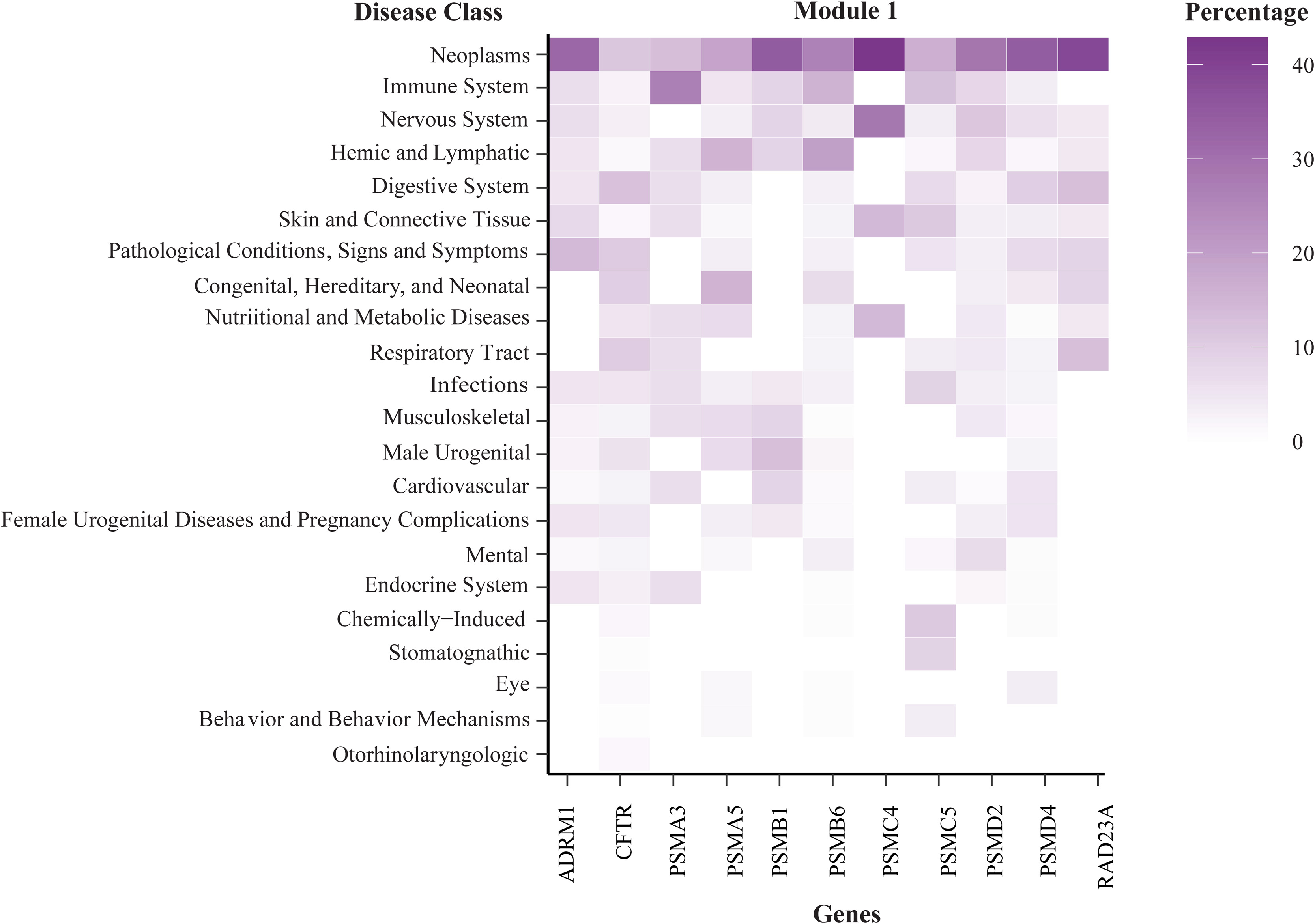

Supplement: Supplementary file 4 — Supplementary Figure 2: Representation of modules-1 genes association with the different disease classes. [file mmc4.jpg]

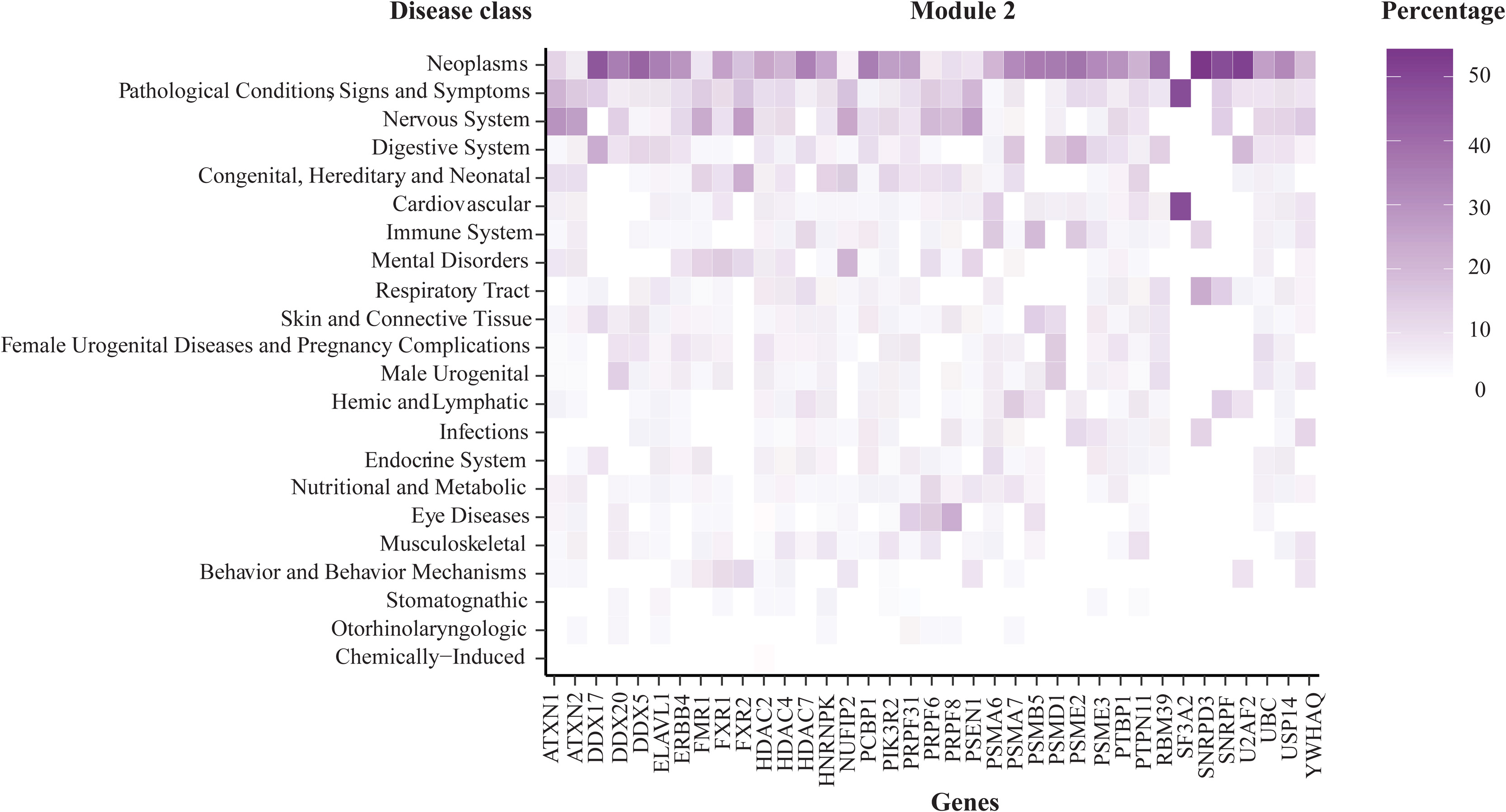

Supplement: Supplementary file 5 — Supplementary Figure 3: Representation of modules-2 genes association with the different disease classes. [file mmc5.jpg]

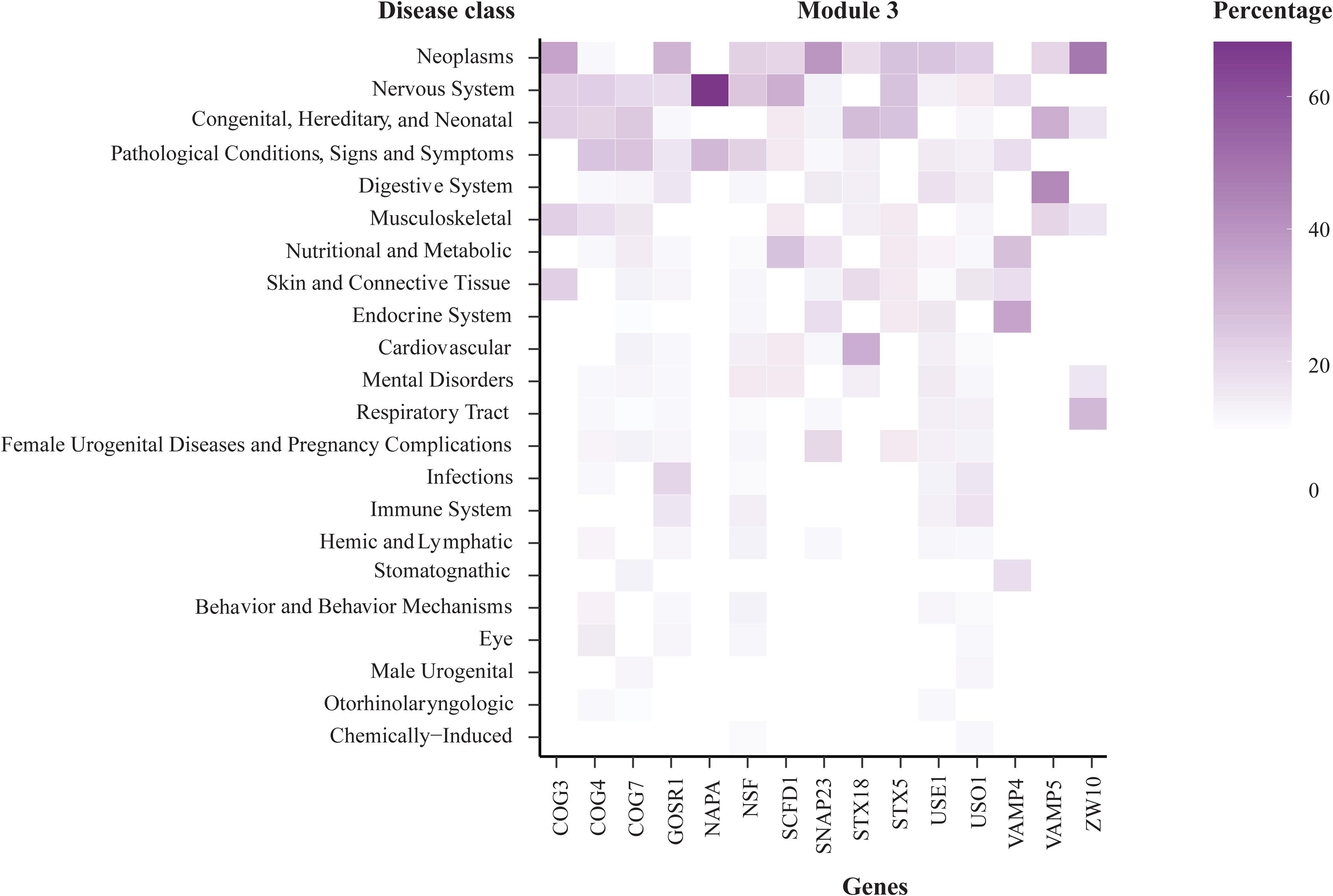

Supplement: Supplementary file 6 — Supplementary Figure 4: Representation of modules-3 genes association with the different disease classes. [file mmc6.jpg]

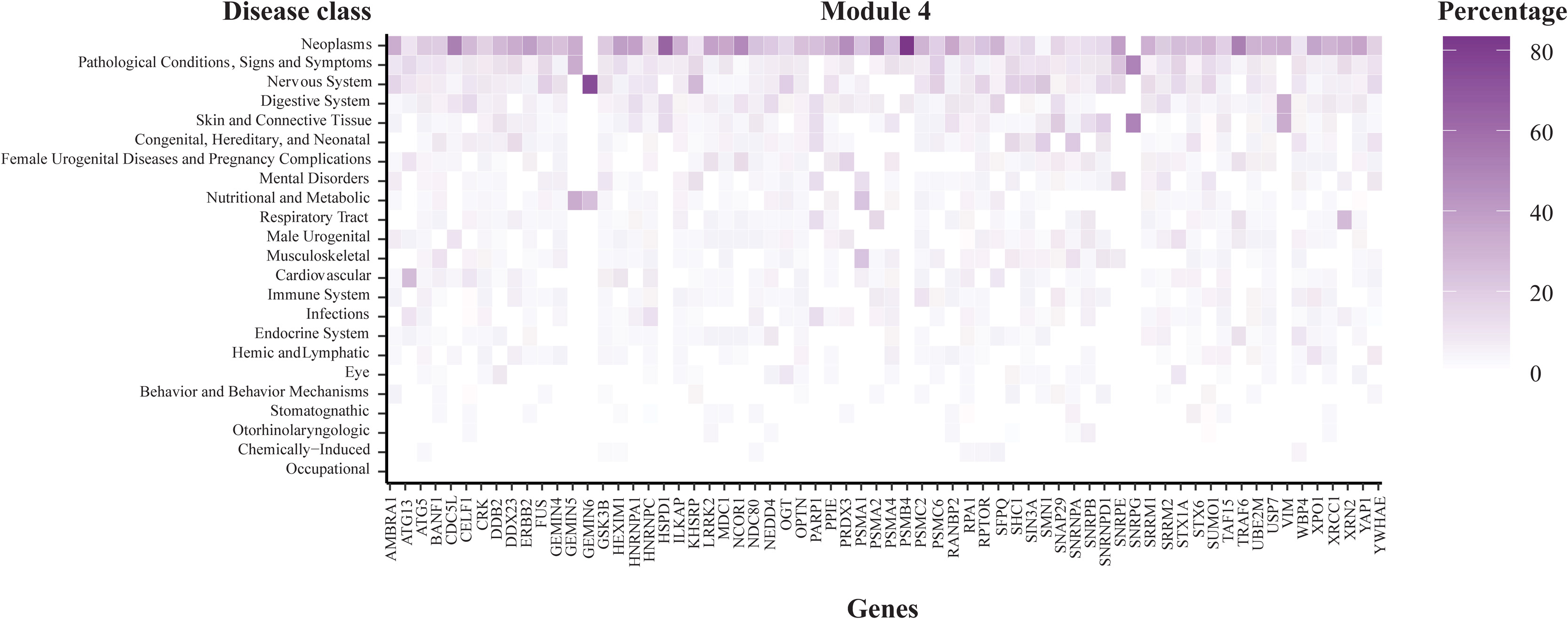

Supplement: Supplementary file 7 — Supplementary Figure 5: Representation of modules-4 genes association with the different disease classes. [file mmc7.jpg]

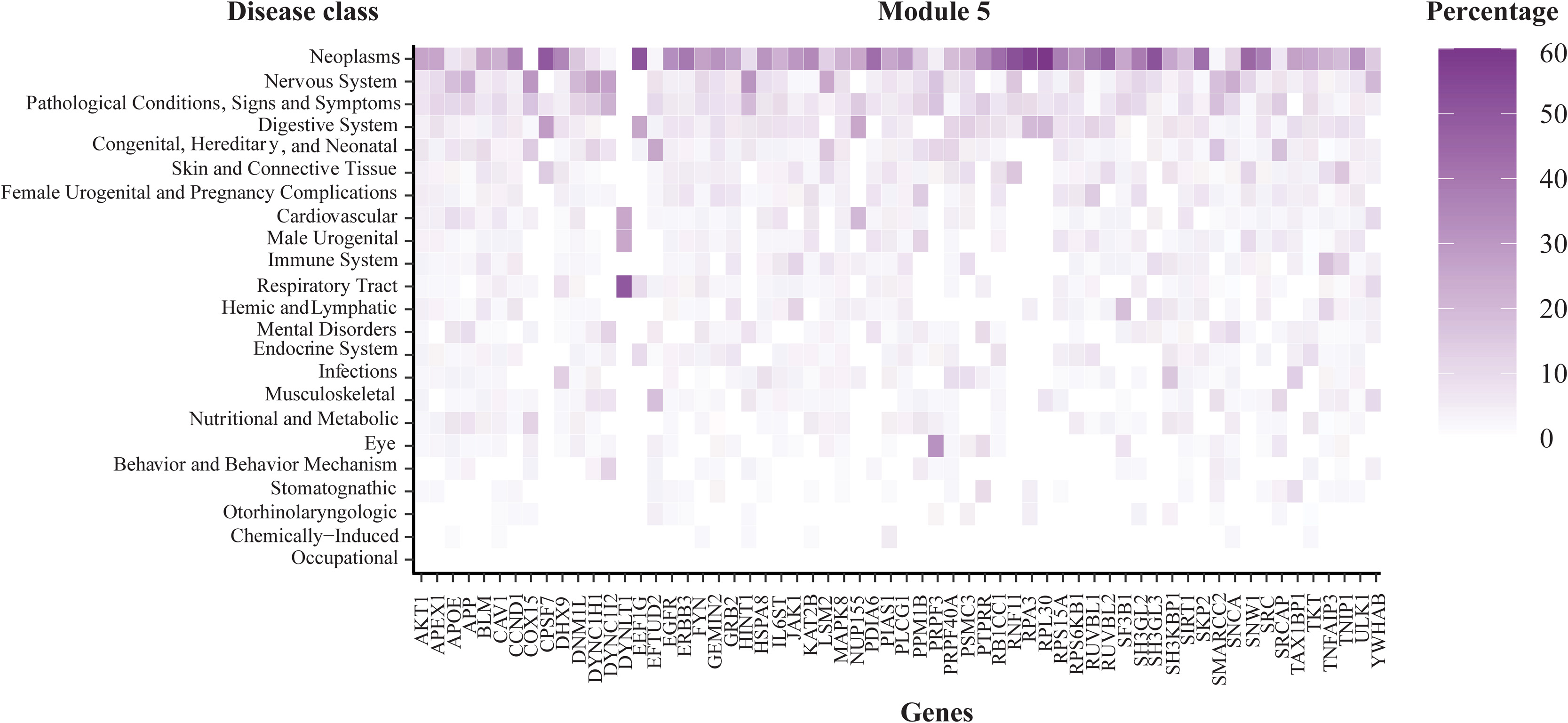

Supplement: Supplementary file 8 — Supplementary Figure 6: Representation of modules-5 genes association with the different disease classes. [file mmc8.jpg]
